# Pristine Photopolymerizable Gelatin Hydrogels: A Low-Cost and Easily Modifiable Platform for Biomedical Applications †

**DOI:** 10.3390/antiox13101238

**Published:** 2024-10-15

**Authors:** Maria Pérez-Araluce, Alessandro Cianciosi, Olalla Iglesias-García, Tomasz Jüngst, Carmen Sanmartín, Íñigo Navarro-Blasco, Felipe Prósper, Daniel Plano, Manuel M. Mazo

**Affiliations:** 1 Biomedical Engineering Program, Enabling Technologies Division, CIMA Universidad de Navarra, 31008 Pamplona, Spain; mparaluce@unav.es (M.P.-A.); oiglesias@unav.es (O.I.-G.); 2 Department for Functional Materials in Medicine and Dentistry, Institute of Functional Materials and Biofabrication, University of Würzburg, 97070 Würzburg, Germany; alessandro.cianciosi@aofoundation.org (A.C.); tomasz.juengst@uni-wuerzburg.de (T.J.); 3 Bavarian Polymer Institute, University of Bayreuth, 95447 Bayreuth, Germany; 4 Department of Pharmaceutical Sciences, Universidad de Navarra, 31008 Pamplona, Spain; sanmartin@unav.es; 5 Department of Chemistry, Universidad de Navarra, 31008 Pamplona, Spain; inavarro@unav.es; 6 Hematology and Cell Therapy Area, Clínica Universidad de Navarra, Instituto de Investigación Sanitaria de Navarra (IdiSNA), 31008 Pamplona, Spain; fprosper@unav.es; 7 Centro de Investigacion Biomedica en Red de Cancer (CIBERONC) CB16/12/00489, 28029 Madrid, Spain; 8 Hemato-Oncology Program, Cancer Division, CIMA Universidad de Navarra, 31008 Pamplona, Spain

**Keywords:** gelatin, hydrogel, biomaterial, selenium, chronic wounds, cardiovascular, tissue engineering

## Abstract

The study addresses the challenge of temperature sensitivity in pristine gelatin hydrogels, widely used in biomedical applications due to their biocompatibility, low cost, and cell adhesion properties. Traditional gelatin hydrogels dissolve at physiological temperatures, limiting their utility. Here, we introduce a novel method for creating stable hydrogels at 37 °C using pristine gelatin through photopolymerization without requiring chemical modifications. This approach enhances consistency and simplifies production and functionalization of the gelatin with bioactive molecules. The stabilization mechanism involves the partial retention of the triple-helix structure of gelatin below 25 °C, which provides specific crosslinking sites. Upon activation by visible light, ruthenium (Ru) acts as a photosensitizer that generates sulphate radicals from sodium persulphate (SPS), inducing covalent bonding between tyrosine residues and “locking” the triple-helix conformation. The primary focus of this work is the characterization of the mechanical properties, swelling ratio, and biocompatibility of the photopolymerized gelatin hydrogels. Notably, these hydrogels supported better cell viability and elongation in normal human dermal fibroblasts (NHDFs) compared to GelMA, and similar performance was observed for human pluripotent stem cell-derived cardiomyocytes (hiPSC-CMs). As a proof of concept for functionalization, gelatin was modified with selenous acid (GelSe), which demonstrated antioxidant and antimicrobial capacities, particularly against *E. coli* and *S. aureus*. These results suggest that pristine gelatin hydrogels, enhanced through this new photopolymerization method and functionalized with bioactive molecules, hold potential for advancing regenerative medicine and tissue engineering by providing robust, biocompatible scaffolds for cell culture and therapeutic applications.

## 1. Introduction

The synthesis and characterization of novel biomaterials in recent years have played a pivotal role in the advancement of tissue engineering, disease modeling, and drug delivery fields. Among various biomaterials, hydrogels have emerged as a prominent option. Hydrogels, possessing a three-dimensional structure with a high water content, can be broadly classified into two categories: synthetic and natural [1]. Synthetic polymers, such as polyethylene glycols (PEG), polyacrylates, and polyvinyl alcohols (PVA), offer tunability but often encounter biocompatibility challenges. In contrast, hydrogels composed of natural polymers like gelatin, collagen, hyaluronic acid, and polysaccharides show enhanced biocompatibility, cell adhesion motifs, and biodegradability, although with some complexity in modification [2].

Gelatin, derived from collagen hydrolysis, a protein abundant in bones, skin, scales, and tendons of bovine and porcine animals, has gained prominence as a biomaterial. Its advantages, including cost effectiveness, availability, biocompatibility, presence of cell adhesion motifs, and biodegradability, have led to its diverse applications and the Food and Drug Administration (FDA) approval for medical use [3]. However, a key drawback is its temperature sensitivity: although it forms gels at room temperature, it dissolves above 25 °C, limiting its application in medical scenarios requiring physiological conditions (37 °C). To address this, various crosslinking methods have been explored, including enzymatic systems with horseradish peroxidase or microbial transglutaminase [4,5]. Additionally, photopolymerizable gelatin modified with methacrylic anhydride (GelMA) has found extensive use in tissue engineering and regenerative medicine [6]. Notably, recent advances in click chemistry have allowed rapid and controlled crosslinking leading to more spatially organized molecular architectures, thus generating more stable gels by covalent modification of gelatin with tetrazine, allyl groups, or norbornene [7,8].

In our study, we have innovatively developed a method for creating stable gels at 37 °C through photopolymerization using pristine gelatin. The absence of gelatin modification simplifies and expedites the hydrogel production process, eliminating the need for solvents and chemical reactions. Furthermore, the use of commercially available medical-grade gelatin circumvents labour-intensive processes like filtration, dialysis, and subsequent lyophilization, thereby avoiding discrepancies between production batches. The stabilization mechanism involves the partial retention of the triple-helix structure of gelatin below 25 °C, which provides specific crosslinking sites. Visible light-induced activation of the photosensitizer ruthenium (Ru) generates sulphate radicals from sodium persulphate (SPS), inducing covalent bonding between tyrosine residues and fixing the triple-helix conformation [9].

The ability to obtain stable hydrogels from pristine gelatin opens avenues for leveraging established methods of modification. Instead of employing functional groups to crosslink the gelatin, any molecule can be used to confer additional properties to our material. As a proof of concept for functionalization, we chose to modify gelatin with selenous acid (GelSe) through a simple, solvent-free reaction, capitalizing on its antioxidant and antimicrobial properties [10,11]. This modification holds promise for applications such as the treatment of chronic wounds and myocardial infarction, where reactive oxygen species play a role in tiling healing towards a pathological situation. Our study introduces a novel approach to obtain hydrogels from pristine gelatin through photopolymerization. We have comprehensively characterized their mechanical properties, swelling ratio, biocompatibility, and their ability to encapsulate different cell types with significant implications for regenerative medicine and tissue engineering as normal human dermal fibroblasts (NHDFs) and cardiomyocytes derived from human pluripotent stem cells (hiPSC-CMs). Additionally, we have successfully developed a new selenated gelatin hydrogel (GelSe) with antioxidant and antimicrobial properties. 

## 2. Materials and Methods

### 2.1. Hydrogel Formation

**Figure 1:**
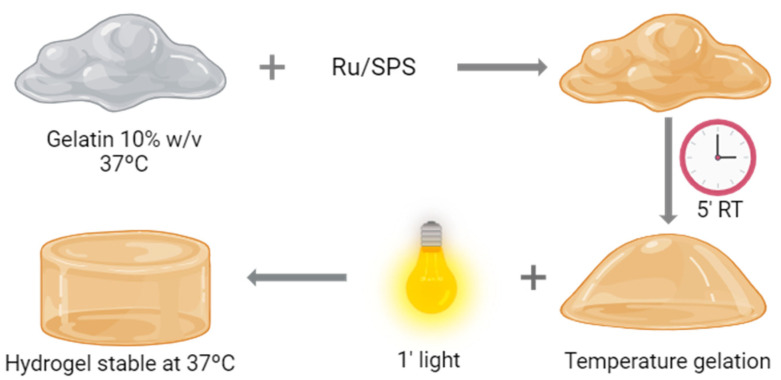
Hydrogel formation. Schematic representation of the polymerization process of pristine gelatin.

Hydrogel formation was performed by photopolymerization; a schematic representation of the process is shown in Figure 1. For this purpose, the combination of Tris(2,2′-bipyridyl)dichlororuthenium(II) hexahydrate (Ru) (Sigma-Aldrich, St. Louis, MO, USA) and sodium persulfate (SPS) (Sigma-Aldrich) was employed as a photosensitizer and an oxidizing agent, respectively. Pristine gelatin (gelatin from porcine skin, 300 g Bloom, Type A, Sigma-Aldrich) was dissolved at a final concentration of 20% in PBS at 37 °C. We diluted the gelatin by half by adding PBS and Ru and SPS to a final concentration of 1 and 10 mM, respectively. This solution is placed at room temperature for 5 min, allowing the gelatin to form a temperature-reversible gel. Once finishing this time, the gelatin with the photoinitiators is placed under a visible light source (30 mW cm^−2^) for 1 min.

### 2.2. Pristine Gelatin and Hydrogel Characterization

#### 2.2.1. Rheological and Photorheological Characterization

The viscoelastic properties of the pristine gelatin hydrogels were measured with an Anton Paar MCR 702 rheometer (Anton Paar, Graz, Austria) with a 25 mm parallel plate geometry, featuring a gap of 500 µm. To limit the solvent evaporation during the experiments, a solvent trap was used. The hydrogel precursors, freshly prepared, were loaded onto the lower plate either at 37 °C or at room temperature (21 °C). The oscillatory measurements were performed with an oscillatory frequency of 10 rad/s and a shear strain of 10%; these values were extracted from the linear viscoeslastic region (LVR). The photorheological analysis was performed using a custom-made set-up obtained by integrating the rheometer with a UV–visible light source (Dr. Hönle, Bluepoint 4, Gräfelfing, Germany) equipped with a flexible light guide. The wavelength spectrum was narrowed using an optical filter within the range of 390 to 500 nm. The glass material of the lower plate facilitated the penetration of UV–visible light to crosslink the precursors with an exposure time of 35 s at a distance of 7.5 cm (light probe to the lower plate). A temperature sweep of the pristine gelatin and its viscosity profile at different concentrations (10, 7.5, and 5%) were also quantified.

#### 2.2.2. Measurement of Pristine Gelatin Hydrogel Autofluorescence from Dityrosine

Cylindrical pristine gelatin hydrogels formed by photopolymerization as described before (light-activated hydrogels) and pristine gelatin with Ru/SPS but without photopolymerization (non-activated hydrogels) (diameter: 6 mm, height: 1 mm) were placed under UV light (Vilber, Collégien, France, VL-215-LC) [9]. As controls, Ru (1 mM) and SPS (10 mM) in PBS and pristine gelatin at 10% *w*/*v* in PBS were employed, with the latter placed at room temperature for 5 min to allow the gelatin to form temperature-reversible gels.

#### 2.2.3. Swelling Ratio

To measure the swelling ratio, cylindrical gelatin hydrogels were formed (diameter: 6 mm, height: 1 mm, N = 12) and incubated at 37 °C in PBS with systematic buffer changes over time. Images were taken, and their diameter was measured with a calliper on days 1, 4, 6, 15, 22, and 30.

### 2.3. Cell Culture and Isolation

All cells were maintained at 37 °C and 5% O_2_ in a humidified incubator (Hera cell 240). All products were purchased from Life Technologies unless otherwise stated.

#### 2.3.1. Normal Human Dermal Fibroblasts (NHDF) Juvenile Foreskin

Normal human dermal fibroblasts (NHDF) juvenile foreskin (PromoCell, Heidelberg, Germany, C-12300) were cultured in PromoCell growth medium supplemented with Fibroblast Growth Factor (FGF) upon 10 ng/mL and 1% penicillin/streptomycin, renewed every two days. Upon confluence, cells were detached with Trypsin/EDTA and subcultured.

#### 2.3.2. Human Induced Pluripotent Stem Cells (hiPSCs) and Cardiac Differentiation

Human iPSCs (WTC11 cell line, a kind gift from Prof. Bruce Conklin, Gladstone Institutes), were cultured in Essential 8 (E8) medium, renewed every day. Upon confluence, they were washed with Dulbecco’s modified phosphate buffered saline solution (DPBS) and detached with 0.5 mM EDTA in PBS. Afterwards, hiPSCs were passaged at a 1:15 ratio in E8 supplemented with the ROCK inhibitor Y27623, onto surfaces coated with 1:180 growth factor reduced Matrigel.

Differentiation to cardiomyocytes (CMs) was based on a biphasic modulation of the Wnt pathway. Once cells became confluent, they were exposed to CHIR 6 μM in RPMI supplemented with B27 without insulin (RPMI + B27-INS) for 24 h, after which medium was changed to RPMI + B27-INS for 48 h. After that, the Wnt inhibitor C59 was added at a final concentration of 2 μM on RPMI + B27-INS medium for 48 h. Cells were cultured in RPMI medium supplemented with full RPMI + B27 medium for 48 h. Once beating had been established, a metabolic-based selection was performed by supplementing no-glucose RPMI with 4 mM lactate. Two rounds of 72 h in this medium, separated by 48 h in RPMI + B27, were performed, and the cells were ready for isolation.

For hiPSC-CM isolation, cultures were washed three times with 0.5 mM EDTA in PBS and incubated in TrypleE for 7–10 min at 37 °C. Then, cells were mechanically disaggregated by pipetting and TrypleE inactivated with RPMI + B27 medium. Prior to use, cells were counted on a Neubauer chamber using trypan blue to evaluate viability before pelleting at 1000 rpm for 10 min.

### 2.4. Material Addition to Cell Culture and Cell Encapsulation

Approximately 80,000 and 10,000 hiPSC-CMs and NHDFs were seeded per well in a 96-well plate, respectively, and after one week, gelatin at 10% *w*/*v* was added for 24 h. GelMA (PhotoGel^®^~95% DOM, #5208 Advanced Biomatrix, Carlsbad, CA, USA) at 5% *w*/*v* was used as control. For cell encapsulation, we made 35 µL hydrogels. For that, 20% of pristine gelatin was diluted in half by adding the cells with medium and Ru/SPS (at 1 and 10 mM, respectively). Hydrogels were exposed to light as previously explained. One million hiPSC-CMs and 100,000 of NHDFs were encapsulated per hydrogel. GelMA was used as a control at a final concentration of 5%, 0.5/5 mM of Ru/SPS, and 1 min of light exposure.

### 2.5. Cytotoxicity Assays: Live/Dead^®^ and Alamar Blue

To assess the cytotoxicity of gelatin and gelatin hydrogel, two assays were conducted: Live/Dead^®^ staining and the Alamar Blue metabolic activity test. GelMA was employed as a control. Live/Dead^®^ staining was performed according to the manufacturer’s instructions. In brief, a solution of 1:500 ethidium homodimer-1 and calcein AM in culture medium was prepared. Subsequently, the culture medium was replaced with this solution and incubated for 45 min at 37 °C. Samples were imaged in a Leica DMIL LED epifluorescence microscope. The Alamar Blue assay is a quantitative method for estimating the viability of cell cultures by measuring their metabolism. For this purpose, a 1:10 Alamar Blue solution in culture medium was prepared, and cells were incubated for 2 to 4 h at 37 °C. Finally, the supernatant was transferred to a new 96-well plate, and absorbance at 570 and 600 nm was measured.

Alamar Blue assays were conducted with NHDFs and hiPSC-CMs seeded in a 96-well plate 24 h after material addition and at days 1, 7, and 14 of cell-encapsulated hydrogels. Live/Dead^®^ staining was performed with NHDFs and hiPSC-CMs encapsulated hydrogels at day 14.

### 2.6. Gelatin Functionalization with Selenium (GelSe)

**Figure 2:**
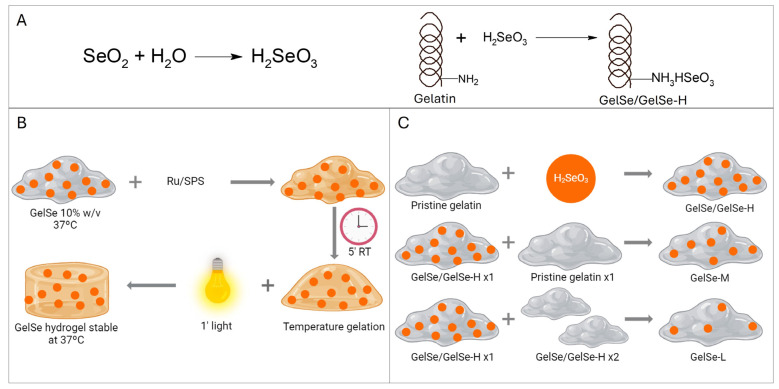
Gelatin functionalization with selenium (GelSe). (**A**) Synthesis reaction of GelSe. (**B**) Schematic representation of GelSe hydrogel formation. (**C**) Schematic representation of the obtention of gelatin with different degree of functionalization (DoF) by mixing GelSe/GelSe-H (high DoF) with pristine gelatin in proportions 1:1 and 1:2 to obtain GelSe-M (medium DoF) and GelSe-L (low DoF), respectively.

Ten grams of porcine gelatin (300 g Bloom, Type A, Sigma-Aldrich) was dissolved in PBS to a final concentration of 10% (*w*/*v*) at 50 °C until the gelatin was fully dissolved. While stirring, 754 mg of selenium dioxide (Sigma-Aldrich) was added to the gelatin solution. The reaction was stirred for 4 h, then dialyzed in 12–14 kDa MWCO dialysis tubing for one week against ddH_2_O to eliminate the unreacted selenium dioxide with regular water exchange. After that, the solution was sterile filtered (0.20 μm) and frozen at −80 °C overnight prior to lyophilization. Three different gelatins were obtained by combining GelSe with varying proportions of pristine gelatin to modify the degree of functionalization (DoF). GelSe obtained in the reaction was designated GelSe with high DoF (GelSe-H or GelSe). GelSe mixed with gelatin in a 1:1 ratio was termed GelSe with medium DoF (GelSe-M), and GelSe mixed with gelatin in a 1:2 ratio was termed GelSe with low DoF (GelSe-L). The reactions are summarized in Figure 2.

### 2.7. Modified Gelatin Characterization

#### 2.7.1. Nuclear Magnetic Resonance (NMR)

^1^*H*-NMR and ^77^*Se*-NMR of GelSe and pristine gelatin were performed using D_2_O as solvent, and NMR spectra were recorded on a Bruker Avance Neo 400 MHz. Chemical shifts were reported in δ_values (ppm).

#### 2.7.2. Atomic Absorption Spectroscopy (AAS)

A Perkin Elmer AAnalyst 800 atomic absorption spectrometer (Perkin Elmer, Hamburg, Germany) equipped with a deuterium lamp for background correction and a flame atomizer was used to carry out the selenium analytical determination. The instrumental parameters were set according to the manual recommendations, and other parameters were optimized. In this regard, (i) a selenium electrodeless discharge lamp was employed as the radiation source operating at 196.0 nm with a current of 290.0 mA and a slit width of 0.2 nm; (ii) an air/acetylene mixture at a flow rate of 17.0 and 2.0 L/min, respectively, was used to generate the flame; and, (iii) burner head height and nebulizer flow rate were set to optimum values at which the maximum absorbance was obtained for the optimization standard solution. 

Matrix-matched calibration selenium standard solutions of 2.5, 5.0, 10.0, and 30 mg/L were prepared by diluting the appropriate volume of standard stock solution 1000 mg/L (Merck, Darmstadt, Germany) in blank gelatin solution containing 0.2% nitric acid solution. To quantify the amount of selenium obtained in the GelSe reaction per gram of gelatin, six portions of GelSe samples (250 mg accurately weighted) were diluted to 25 mL with 0.2% nitric acid solution. An internal quality control (5.2 mg/L) was assessed in every series of samples to check the reproducibility and accuracy of the measurements (n = 3, 5.3 ± 0.3 mg/L). In addition, a recovery study spiking at three different concentrations (2.0, 4.0, and 6.0 mg/L) within the linear calibration range provided satisfactory findings between 98 and 103%, guaranteeing accurate and precise determination of selenium in gelatin samples.

### 2.8. Antioxidant Properties of GelSe

We aimed to study the antioxidant capacity of the hydrogel by performing a DPPH assay using ascorbic acid as a positive control. A DPPH solution was prepared by dissolving DPPH in acetic acid at a final concentration of 0.5 mg/mL. GelSe-H was dissolved at 20% *w*/*v* in acetic acid. In a 96-well plate, 100 µL of the DPPH solution was combined with 100 µL of the GelSe-H solution. A standard was prepared by combining 100 µL of the DPPH solution with 100 µL of acetic acid.

As a control, ascorbic acid was dissolved in acetic acid at a concentration of 0.23 mg/mL. This concentration was chosen as it is equivalent to the selenium concentration found in GelSe-H at 10% *w*/*v* according to data obtained from AAS. As a positive control, we used ascorbic acid at a concentration of 2 mg/mL, as this is a sufficiently high concentration to achieve total inhibition of DPPH.

The plate was incubated in complete darkness for 30 min. Absorbance was then measured at 517 nm. The percentage of antioxidant capacity of GelSe was calculated using the following formula:

% of antioxidant activity = [(AC − AS) ÷AC] × 100

where AC—absorbance of the DPPH control; AS—absorbance of the samples

The antioxidant capacity of GelSe relative to the positive control (ascorbic acid at 2 mg/mL) was calculated by dividing the above value obtained from GelSe by that of ascorbic acid at 2 mg/mL.

### 2.9. Antibacterial Properties of GelSe

The antibacterial properties of GelSe/GelSe-H, GelSe-M, and GelSe-L were investigated against *Escherichia coli* (*E. coli*) and *Staphylococcus aureus* (*S. aureus*) as representative Gram-negative and Gram-positive bacteria, respectively. The bacterial strains were kindly provided by the Department of Microbiology and Parasitology of the University of Navarra, cultured on tryptic soy agar (TSA) plates and maintained at 4 °C until use.

For each material, a 20% *w*/*v* stock solution was prepared in tryptic soy broth (TSB). From this stock solution, five different concentrations were prepared: 13.33% (GelSe.1), 10% (GelSe.2), 6.67% (GelSe.3), 5% (GelSe.4), and 3.33% *w*/*v* (GelSe.5). This concentration scheme was similarly applied to GelSe-M and GelSe-L, resulting in GelSe-M.1 to GelSe-M.5 and GelSe-L.1 to GelSe-L.5.

In a 96-well round-bottom plate, 300 µL of each of the aforementioned concentrations was added to the corresponding wells. Simultaneously, 1 million *E. coli* bacteria were inoculated in each well. The plate was then incubated overnight at 37 °C. Bacterial growth in each well was assessed the following day to determine the Minimum Inhibitory Concentration (MIC). Subsequently, 5 µL of the samples from each well was collected and cultured overnight at 37 °C on an agar plate to determine the Minimum Bactericidal Concentration (MBC). The same procedure was repeated for *S. aureus*.

### 2.10. Biocompatibility Properties of GelSe

For the biocompatibility testing of GelSe, NHDFs (10,000/well) were seeded onto 96-well plates. After two days of culture, GelSe-H and GelSe-M were added at the same concentrations as those used in the antibacterial properties assay. Specifically, five concentrations were tested for each material: GelSe.1, GelSe.2, GelSe.3, GelSe.4, and GelSe.5, all reaching a final volume of 300 µL per well. GelSe-L was excluded from testing due to its lack of bactericidal activity. The objective of this study was to ascertain whether the observed bactericidal activity in the previous assay stemmed from compound toxicity. Following the addition of materials to the cell cultures, they were incubated for 24 h at 37 °C, after which an Alamar Blue AND LIVE/DEAD^®^ assay was performed.

### 2.11. Statistical Analyses

Statistical analyses were performed using GraphPad 5. A normal distribution of the data was assumed, and an unpaired *t*-test was applied for comparisons between two groups. 

## 3. Results

### 3.1. Pristine Gelatin and Hydrogel Characterization

#### 3.1.1. Rheological and Photorheological Characterization

To investigate the properties and potential applications of pristine gelatin, we conducted a series of tests to characterize its viscoelastic behavior and photocrosslinking efficacy. In this study, we aimed to determine the influence of gelatin concentration on viscosity and gel formation, as well as to investigate the effects of photopolymerization on the structure and stability of the resulting hydrogels.

**Figure 3:**
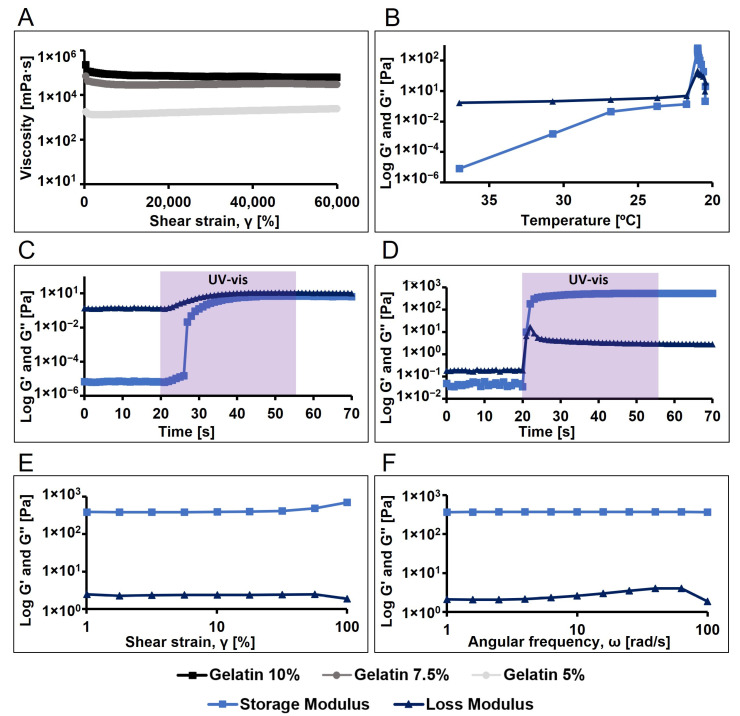
Rheological and photorheological tests (n = 3, 10 rad/s oscillation frequency, 10% shear strain) for the analysis of the viscosity and the viscoelastic properties of the pristine gelatin and polymerize pristine gelatin. (**A**) Viscosity profile of pristine gelatin at different concentrations (10, 7.5 and 5% *w*/*v*). (**B**) Temperature sweep of pristine gelatin. (**C**) Time sweep of gelatin at 37 °C (light activated at 20 s, 35 s exposure time, 7.5 cm light probe-to-sample distance). (**D**) Time sweep of gelatin previously incubated for 5 min at 21 °C (light activated at 20 s, 35 s exposure time, 7.5 cm light probe-to-sample distance). (**E**) Amplitude sweep of pristine gelatin hydrogel. (**F**) Frequency sweep of pristine gelatine hydrogel.

We began by evaluating the viscosity of gelatin dissolved in PBS at different concentrations (10%, 7.5%, and 5% *w*/*v*), observing an expected increase in viscosity with higher concentrations (Figure 3A). The 10% concentration was selected due to its higher viscosity, suggesting better thermoreversible crosslinking at room temperature. Additionally, we investigated the gel formation of pristine gelatin dissolved at 10%, observing gel formation below 22 °C (Figure 3B). Subsequently, to characterize the viscoelastic properties and establish the linear viscoelastic region (LVR) of 10% pristine gelatin, amplitude and frequency sweep tests were conducted, enabling us to select an oscillatory frequency of 10 rad/s and a shear strain of 10% for the oscillatory analysis as time and temperature sweep.

The photopolymerization of pristine gelatin was analysed at both 37 °C and 21 °C. It was observed that when gelatin was mixed with Ru and SPS (1 and 10 mM, respectively) and light was applied, a viscous liquid was obtained but no hydrogel formed, as the loss modulus (G″) remained higher than the storage modulus (G′) (Figure 3C). However, when pristine gelatin with photoinitiators was allowed to gel by temperature (21 °C), applying light resulted in the formation of a stable gel (Figure 3D). Additionally, we examined the viscoelastic properties of the formed hydrogel using amplitude and frequency sweep tests (Figure 3E,F).

#### 3.1.2. Measurement of Pristine Gelatin Hydrogel Autofluorescence from Dityrosines

**Figure 4:**
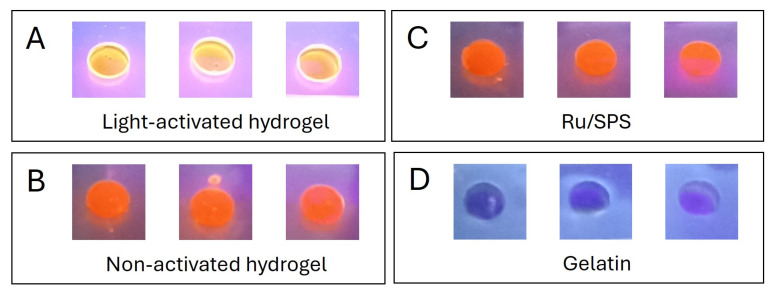
Autofluorescence of dityrosine groups in response to UV light exposure. (**A**) Light-activated pristine gelatin hydrogels. (**B**) Non-activated pristine gelatin hydrogels. (**C**) Ru/SPS 1/10 mM solution in PBS. (**D**) Pristine gelatin 10% *w*/*v* in PBS.

The stabilization mechanism of the pristine gelatin hydrogels involves the partial retention of the triple-helix structure of gelatin below 25 °C, which provides specific sites for crosslinking. Upon activation by visible light, ruthenium (Ru) acts as a photosensitizer, generating sulfate radicals from sodium persulfate (SPS). This process induces covalent bonding between tyrosine residues, forming dityrosine groups, effectively “locking” the triple-helix conformation. To confirm the formation of dityrosine crosslink bonds in these light-activated hydrogels, we measured the autofluorescence intensity of the samples when exposed to UV light (Figure 4). Previous studies have shown that stable dityrosine bonds contain phenolic groups that emit fluorescence upon UV irradiation [9,12]. The autofluorescence observed in the light-activated gels (Figure 4A), and its absence in the non-activated ones (Figure 4B) and the controls (Figure 4C,D), demonstrates that the polymerization process occurred via this mechanism.

#### 3.1.3. Swelling Ratio

**Figure 5:**
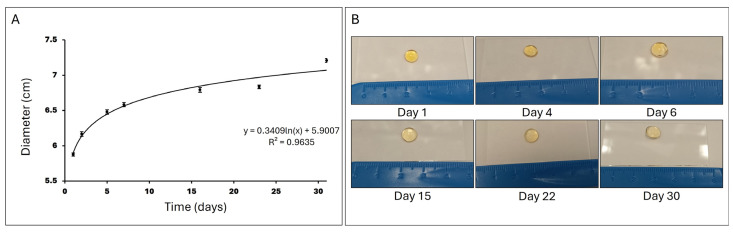
Swelling ratio. (**A**) Representation of the hydrogel diameter in cm after incubation in PBS at 37 °C over 30 days (N = 12). (**B**) Images showing the evolution of the hydrogel incubated in PBS at 37 °C over 30 days.

When hydrogels are immersed in PBS at 37 °C, they initially absorb the liquid rapidly, leading to a swift increase in diameter. As time progresses, the rate of PBS absorption decreases, and the hydrogel approaches an equilibrium state where it can no longer absorb additional PBS due to the elastic resistance of the polymer network and the interaction with the solvent. This behavior is described in Figure 5 by a logarithmic function, where the growth is rapid at the beginning and then stabilizes. 

### 3.2. Material Addition to Cell Culture

**Figure 6:**
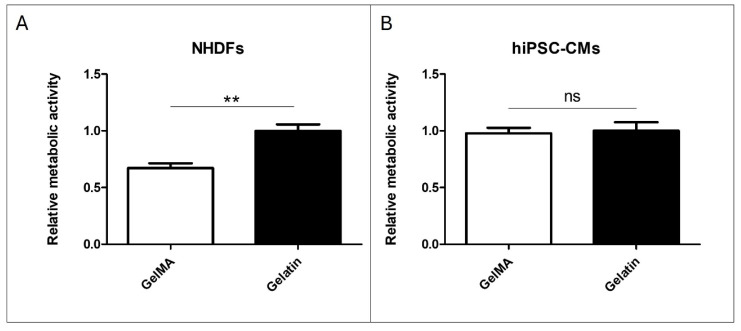
Material addition to cell culture. (**A**) Alamar Blue assay of pristine gelatin and GelMA 10% *w*/*v* addition to NHDFs cultured in a 96-well plate. (**B**) Alamar Blue assay of pristine gelatin and GelMA 10% *w*/*v* addition to hiPSC-CMs cultured in a 96-well plate. (N = 3, unpaired *t*-test, ** *p* < 0.005, ns: no significant differences).

The results of adding pristine gelatin and GelMA to NHDF culture demonstrate that cell viability with GelMA is 40% lower than those cultivated with pristine gelatin (Figure 6A). However, in the comparison made with hiPSC-CM, no significant differences were found between the addition of pristine gelatin and GelMA to the cell culture (Figure 6B). These findings suggest differential effects of GelMA on NHDF and hiPSC-CM cultures, highlighting the need for further investigation into the underlying mechanisms. 

### 3.3. Cell Encapsulation

**Figure 7:**
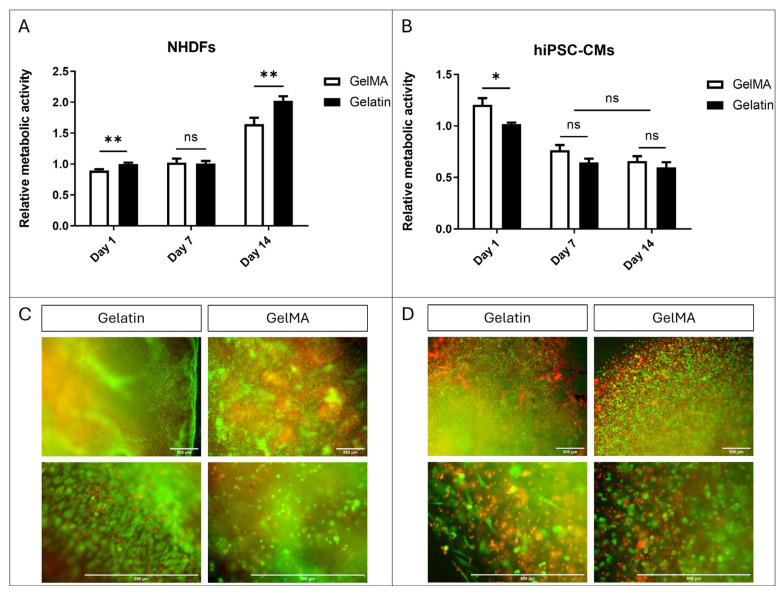
Cell encapsulation. (**A**) Alamar Blue assay of NHDFs encapsulated within pristine gelatin and GelMA 10% *w*/*v*. (**B**) Alamar Blue assay of hiPSC-CMs encapsulated within pristine gelatin and GelMA 10% *w*/*v*. (N = 3, unpaired *t*-test, * *p* < 0.05, ** *p* < 0.005, ns = no significative differences). (**C**) Fluorescence images of Live/Dead^®^ assay of NHDFs encapsulated within pristine gelatin and GelMA 10% *w*/*v*. (**D**) Fluorescence images of Live/Dead assay of hiPSC-CMs encapsulated within pristine gelatin and GelMA 10% *w*/*v*. Live cells produced green fluorescence and dead cells showed red fluorescence.

The viability results of fibroblast and hiPSC-CM encapsulation in GelMA and pristine gelatin are depicted in Figure 7. Regarding NHDFs, Alamar Blue assay results show slightly better viability in gelatin compared to GelMA. This viability is sustained at day 7 without significant differences between fibroblasts encapsulated in both materials. However, by day 14, we observe an increase in viability significantly greater in those within gelatin gels (Figure 7A). Furthermore, Live/Dead^®^ images taken on day 14 also demonstrate that cells are capable of acquiring a more elongated morphology within gelatin gels while remaining rounded within GelMA gels (Figure 7C). These results suggest that NHDFs are more proficient in remodelling the structure of pristine gelatin hydrogels than GelMA.

Regarding hiPSC-CMs, a decrease in viability is observed at day 7 in both cells encapsulated in pristine gelatin and GelMA. However, this viability is maintained at day 14 without significant differences between GelMA and gelatin (Figure 7B). The results of the Live/Dead^®^ assay support those obtained in AlamarBlue, showing a higher proportion of live cells. Furthermore, interestingly, we also observe that hiPSC-CMs can elongate within pristine gelatin hydrogels while remaining rounded in GelMA (Figure 7D). The lack of increased viability, despite the cells’ ability to remodel the hydrogel structure, is probably due to their inability to divide.

The capacity of pristine gelatin hydrogel to support cellular growth confirms its potential for cell culture applications, particularly in the areas of regenerative medicine and tissue engineering. This observation underscores its suitability as a scaffold material for facilitating tissue regeneration and promoting the development of advanced therapeutic strategies. Additionally, the advantage of cells being able to remodel and elongate within pristine gelatin hydrogels further highlights its utility in these fields, providing opportunities for the creation of functional tissues and enhancing biological response in three-dimensional environments.

### 3.4. Gelatin Functionalization with Selenium (GelSe)

**Figure 8:**
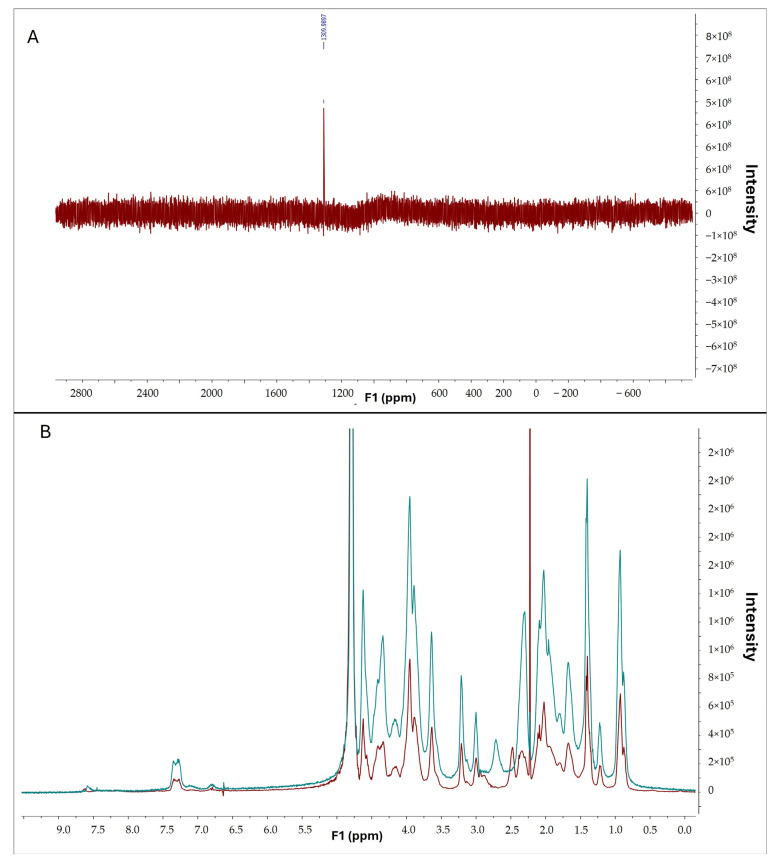
(**A**) ^77^*Se*-NMR of GelSe. (**B**) ^1^*H*-NMR of pristine gelatin (blue) and GelSe (red).

To confirm the interaction between selenous acid and gelatin, selenium and proton nuclear magnetic resonance spectroscopy were conducted. The presence of selenium in gelatin was confirmed by the observed peak in the selenium spectrum (Figure 8A). Furthermore, comparative analysis of ^1^H-NMR spectra of pristine gelatin (in red) and gelatin modified with selenium (in blue) revealed discernible differences (Figure 8B), indicative of a successful chemical reaction between selenous acid and gelatin. 

### 3.5. Atomic Absorption Spectroscopy (AAS)

We performed AAS to determine the accurate selenium concentration per gram of gelatin. The result we obtained indicated the presence of 2.3 ± 0.3 mg of selenium per gram of gelatin in GelSe-H (n = 6). Once this result was obtained, we were able to calculate the amount of selenium in GelSe-M and GelSe-L. GelSe-M, being composed of a 1:1 mixture of GelSe/gelatin, presented a concentration of 1.15 mg Se/g gelatin. Meanwhile, GelSe-L, being composed of a 1:2 mixture of GelSe/gelatin, presented a concentration of 0.57 mg Se/g gelatin. The selenium content in GelSe-M and GelSe-L was also corroborated by the AAS method.

### 3.6. Antioxidant Properties of GelSe

**Figure 9:**
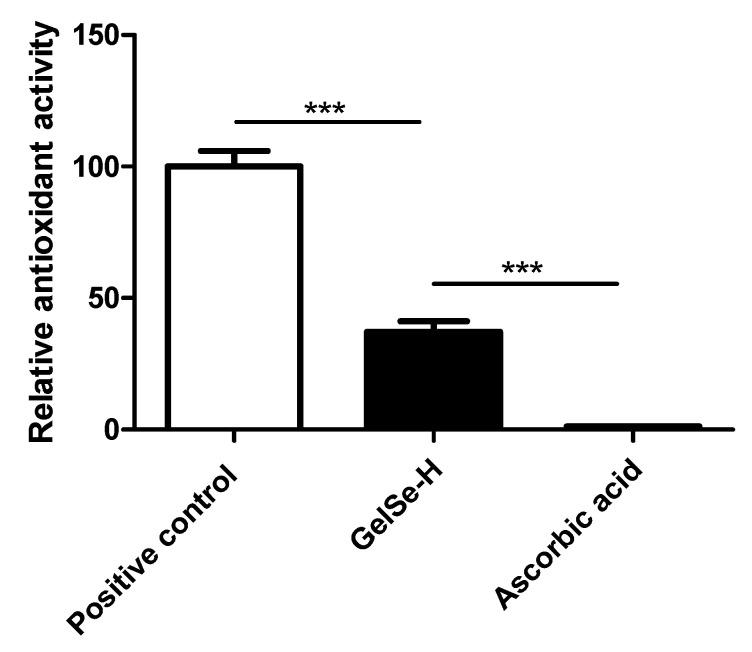
Antioxidant properties of GelSe. DPPH analysis of GelSe-H. Results were calculated relative to a positive control (ascorbic acid 2 mg/mL). The “Ascorbic acid” result is the antioxidant capacity of ascorbic acid at 0.023 mg/mL, the equivalent concentration of selenium found in GelSe-H. (N = 3, unpaired *t*-test, *** *p* < 0.001).

The results of the DPPH assay (N = 3) demonstrated the antioxidant capacity of the novel selenium-enriched hydrogel, GelSe. The findings revealed that GelSe-H exhibited an antioxidant capacity of 37.34% relative to the positive control (ascorbic acid at 2 mg/mL). However, the GelSe-H antioxidant capacity was significantly higher compared to ascorbic acid at an equivalent concentration as the selenium found in GelSe. (Figure 9). 

### 3.7. Antibacterial Properties of GelSe

**Figure 10:**
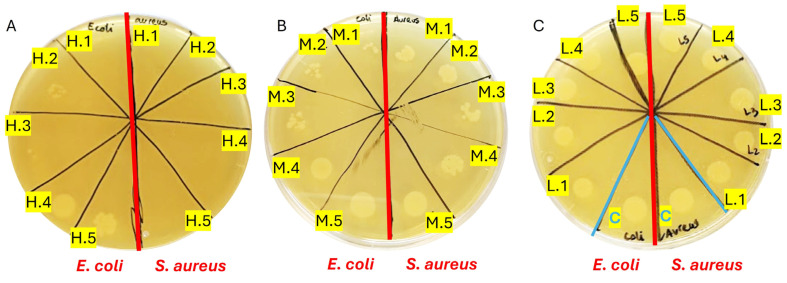
Antibacterial properties of GelSe against *Escherichia coli* (*E. coli*) and *Staphylococcus aureus* (*S. aureus*). (**A**) Antibacterial properties of GelSe/GelSe-H. Agar plate culture of a sample from a 96-well plate containing 1 million bacteria of *E. coli* (left) and *S. aureus* (right), treated with five different concentrations of GelSe (GelSe-H.1 (13%), H.2 (10%), H.3.3 (6.67%), H.4 (5%), and H.5 (3.33% *w*/*v*)). (**B**) Antibacterial properties of GelSe-M.1 to GelSe-M.5 with *E. coli* (left) and *S. aureus* (right). (**C**) Antibacterial properties of GelSe-L.1 to GelSe-L.5 with *E. coli* (left) and *S. aureus* (right), including a control for each bacterial strain using pristine gelatin.

The results demonstrated bactericidal activity against both *Escherichia coli* (*E. coli*) and *Staphylococcus aureus* (*S. aureus*) initiating at GelSe-M.3 (Figure 10B), indicating the minimum inhibitory concentration (MIC) for both bacterial strains. Below this selenium concentration, bacterial growth resembled that of the controls (Figure 10A). Complete inhibition of *S. aureus* growth was observed from GelSe.5, signifying the minimum bactericidal concentration (MBC) specific to *S. aureus*. Conversely, for *E. coli*, such complete inhibition was evident only at GelSe.1 (Figure 10C), representing the MBC for *E. coli*. The activity observed from GelSe-M.3 suggests the existence of a critical concentration threshold. Notably, variability in sensitivity between *E. coli* and *S. aureus* hints at potential specific biomedical applications. 

### 3.8. Biocompatibility Properties of GelSe

**Figure 11:**
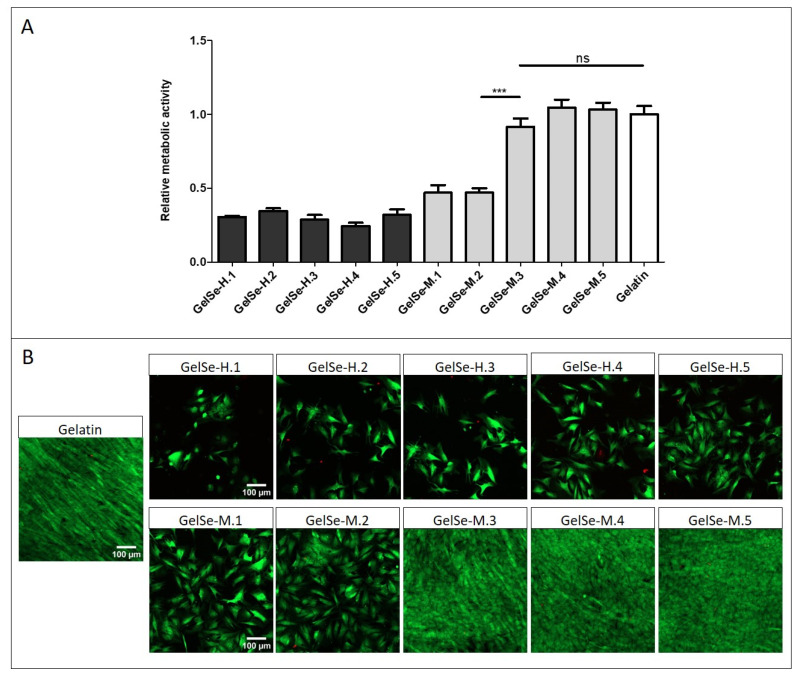
Biocompatibility properties of different concentrations of GelSe-H and GelSe-M added to NHDFs. (**A**) Alamar blue assay (N = 3, unpaired *t*-test, *** *p* < 0.001, ns = no significative differences). (**B**) Fluorescence images of Live/Dead^®^ assay. Live cells produced green fluorescence and dead cells showed red fluorescence. Scale bars are 100 µm.

To further the applicability of the GelSe formulations, we analysed their potential toxicity with NHDF. The study results indicate that all concentrations of GelSe-H demonstrate biocompatibility below 40% when compared to pristine gelatin. GelSe-M exhibits a slightly higher biocompatibility. Specifically, the higher concentrations of GelSe-M (GelSe-M.1 and M.2) show cell viability around 50% compared to pristine gelatin, while GelSe-M.3, M.4, and M.5 exhibit cell viability similar to pristine gelatin (Figure 11). In relation to the previous antibacterial study, these results reflect that GelSe-M.3 demonstrates specific antibacterial activity, as evidenced by a reduction in bacterial growth compared to the pristine gelatin control, while maintaining similar biocompatibility.

## 4. Discussion

The objective of this study was to develop and characterize a novel biomaterial based on gelatin hydrogels for applications in regenerative medicine and tissue engineering. Initially, research focused on cell therapy, which revealed limitations due to the lack of an adequate extracellular matrix (ECM) that mimics the natural tissue microenvironment. The ECM provides not only structural support but also crucial biochemical and biomechanical signals for cell differentiation and proliferation [13]. To address these limitations, we concentrated our efforts on gelatin hydrogels due to their high biocompatibility and ability to form highly hydrated three-dimensional networks, beneficial for 3D cell culture. Additionally, gelatin contains cell adhesion motifs such as RGD (arginine–glycine–aspartic acid) sequences that can be recognized by cell integrins, allowing cell adhesion, proliferation, and differentiation.

The primary challenge of using gelatin lies in its thermal properties. Naturally, gelatin forms hydrogels at temperatures below 25 °C but dissolves at physiological temperature (37 °C). Traditionally, it has been considered necessary to modify gelatin covalently to induce crosslinking and thus maintain the hydrogel structure at 37 °C. However, these modification methods often involve harsh chemical conditions, such as the use of organic solvents or high temperatures, which can degrade the gelatin structure and reduce its biocompatibility.

In this study, we propose a novel method to stabilize gelatin hydrogels at 37 °C without the need for traditional covalent modifications. The stabilization of gelatin hydrogels through photopolymerization using a photosensitizer (Ru) and an oxidizing agent (SPS) is based on the interplay between the temperature-dependent structural properties of gelatin and the radical-mediated crosslinking reactions initiated by Ru and SPS. Gelatin, derived from collagen, partially retains the triple-helix structure when cooled below 25 °C. This conformational change enhances the potential for specific crosslinking sites, as the triple helix brings the gelatin chains into closer proximity. At physiological temperatures, these helical structures tend to disorganize, returning gelatin to a liquid state [14].

In our study, we exploited the natural presence of tyrosine residues in gelatin, which serve as reaction sites for the crosslinking. Upon activation by visible light, Ru acts as a photosensitizer, transferring electrons to SPS, which generates sulphate radicals. These radicals induce covalent bonding between tyrosine residues [9], stabilizing the hydrogel matrix. The photopolymerization process effectively “locks” or “fixes” the triple-helix conformation, reinforcing the gelatin matrix.

As demonstrated by photorheometry, a 10% *w*/*v* gelatin solution containing Ru and SPS at a concentration of 1 and 10 mM, respectively, was allowed to gel at room temperature (21 °C) for 5 min before applying light for 1 min. This process resulted in the formation of a stable hydrogel that remained intact even at physiological temperatures (37 °C), providing a significant improvement over pristine gelatin, which typically dissolves under these conditions.

Once the polymerisation conditions were established, we proceeded to study the mechanical properties of the formed hydrogel through oscillatory rheological measurement tests and the swelling ratio. These results demonstrated that we obtained a stable hydrogel. The swelling ratio increasing logarithmically over time indicates that the rate of water absorption is initially rapid but gradually slows down, achieving equilibrium. These results are crucial for understanding the hydrogel’s dynamics and its practical applications in biomedical fields such as controlled drug release and tissue engineering.

Although the biocompatibility of gelatin is well documented [15], we aimed to study the biocompatibility of the hydrogel with cell cultures. One of the most developed areas for using hydrogels is in the treatment of chronic wounds [16], so we tested whether our hydrogel could also be used for this purpose by studying its biocompatibility with dermal fibroblasts (NHDFs). Cardiac tissue engineering is a less developed field than chronic wound treatment but is crucial, as myocardial infarction is the leading cause of death worldwide, and no effective curative treatments currently exist [17,18]. Therefore, we considered it interesting to study the biocompatibility of the hydrogel with cardiomyocytes derived from hiPSCs (hiPSC-CMs). In both cases, we used GelMA as a control, as it is the most widely used gelatin-based hydrogel in tissue engineering [6,19]. GelMA is modified with methacrylic anhydride to polymerize upon adding photoinitiators and applying light. For dermal fibroblasts, we found that the biocompatibility of gelatin decreases when modified to obtain GelMA, whereas with hiPSC-CMs, we observed similar viability between both types of hydrogels. These results indicate that our new method of gelatin polymerisation offers advantages over current methods, as it eliminates the synthesis process and does not decrease cell viability. The differential effects observed between NHDFs and hiPSC-CMs suggest that the interaction mechanisms between cells and the gelatin matrix need further exploration. Additionally, the long-term stability and in vivo biocompatibility of these hydrogels need to be evaluated in future studies to confirm their practical applicability.

We further tested whether the hydrogels could encapsulate cell cultures, which is fundamental in regenerative medicine and tissue engineering. We observed that in both cases, NHDF and hiPSC-CM cells could remodel the hydrogel, stretch, and adopt an elongated conformation within it, maintaining good viability.

In addition to the previously mentioned advantages of the developed gelatin polymerisation system, one of the primary benefits of not needing to modify the gelatin with a polymerising agent is that we can tailor it to acquire the desired biological activity.

In this study, as a proof of concept, we modified gelatin with selenous acid via a straightforward reaction without the use of organic solvents, resulting in GelSe. It was confirmed that the reaction had taken place using ^1^*H*-NMR and ^77^*Se*-NMR, and the degree of modification of the gelatin with selenous acid was calculated using atomic absorption spectroscopy. Selenium is an essential trace element that plays a crucial role in various biological functions. Its antioxidant, anti-inflammatory, and antibacterial properties have been shown to be beneficial for improving cognitive function, preventing cardiovascular diseases, and supporting the immune system, amongst other benefits [20,21,22,23].

**Figure 12:**
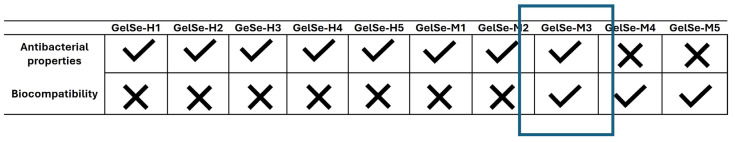
Comparative table of GelSe-H and GelSe-M showing their antibacterial and biocompatibility properties. GelSe-M.3 is proposed as the best candidate, as it demonstrates good cell viability and antibacterial properties. X: not bactericidal/not biocompatible; ✓: bactericidal/biocompatible.

Modifying gelatin with selenium allows us to maintain photopolymerization conditions to form stable gels. The antioxidant capacity of GelSe was studied using the DPPH assay. This assay enabled us to conclude that the selenium molecules bound to the gelatin exhibit a high antioxidant capacity, outstandingly superior to that of equivalent amounts of ascorbic acid. Preliminary studies on the antibacterial and biocompatibility properties of GelSe have been conducted. The antibacterial properties of GelSe were tested against *E. coli* and *S. aureus* by culturing these bacteria with gelatin modified with different proportions of selenium. Simultaneously, we also assessed cell culture viability using NHDFs. The results of both experiments allowed us to determine the optimal degree of selenium modification required to achieve a gelatin with good cell viability while retaining antibacterial properties (Figure 12).

While these studies remain somewhat limited, they provide valuable preliminary insights into the potential of GelSe. Future work will focus on expanding both the antibacterial and biocompatibility characterisations, particularly by encapsulating cells within GelSe hydrogel. This would allow for a more comprehensive understanding of the material’s interactions with cells and its full cytocompatibility profile. Despite these limitations, the current findings offer important information on the balance between antimicrobial activity and cytocompatibility, which is crucial for the further development of GelSe-based materials for biomedical applications since the development of antioxidant and/or antibacterial biomaterials has garnered significant interest in recent years [24,25,26].

## 5. Conclusions

This study successfully developed a novel method to stabilize gelatin hydrogels at physiological temperatures without harsh chemical modifications. The resulting hydrogels demonstrated excellent biocompatibility, mechanical stability, and potential for encapsulating cell cultures. Furthermore, modifying gelatin with selenium enhanced its antioxidant and antibacterial properties while maintaining cell viability. These findings highlight the potential of our gelatin-based hydrogels for various biomedical applications, including regenerative medicine, tissue engineering, and chronic wound and cardiovascular disease treatment. Future work should focus on in vivo studies and the long-term performance of these hydrogels in clinical settings to further validate their applicability and efficacy.

## Data Availability

Dataset available on request from the authors.
